# “Determining the efficacy of a machine learning model for measuring periodontal bone loss”

**DOI:** 10.1186/s12903-023-03819-w

**Published:** 2024-01-17

**Authors:** Diego Cerda Mardini, Patricio Cerda Mardini, Daniela Paz Vicuña Iturriaga, Duniel Ricardo Ortuño Borroto

**Affiliations:** 1 Universidad de los Andes, Chile, Facultad de Odontología, Santiago, Chile; 2 MindsDB, San Francisco, USA

**Keywords:** Artificial intelligence, Machine learning, Neural networks, Periodontal bone loss, Periodontitis

## Abstract

**Background:**

Considering the prevalence of Periodontitis, new tools to help improve its diagnostic workflow could be beneficial. Machine Learning (ML) models have already been used in dentistry to automate radiographic analysis.

**Aims:**

To determine the efficacy of an ML model for automatically measuring Periodontal Bone Loss (PBL) in panoramic radiographs by comparing it to dentists.

**Methods:**

A dataset of 2010 images with and without PBL was segmented using Label Studio. The dataset was split into *n* = 1970 images for building a training dataset and *n* = 40 images for building a testing dataset. We propose a model composed of three components. Firstly, statistical inference techniques find probability functions that best describe the segmented dataset. Secondly, Convolutional Neural Networks extract visual information from the training dataset. Thirdly, an algorithm calculates PBL as a percentage and classifies it in stages. Afterwards, a standardized test compared the model to two radiologists, two periodontists and one general dentist. The test was built using the testing dataset, 40 questions long, done in controlled conditions, with radiologists considered as ground truth. Presence or absence, percentage, and stage of PBL were asked, and time to answer the test was measured in seconds. Diagnostic indices, performance metrics and performance averages were calculated for each participant.

**Results:**

The model had an acceptable performance for diagnosing light to moderate PBL (weighted sensitivity 0.23, weighted F1-score 0.29) and was able to achieve real-time diagnosis. However, it proved incapable of diagnosing severe PBL (sensitivity, precision, and F1-score = 0).

**Conclusions:**

We propose a Machine Learning model that automates the diagnosis of Periodontal Bone Loss in panoramic radiographs with acceptable performance.

**Supplementary Information:**

The online version contains supplementary material available at 10.1186/s12903-023-03819-w.

## Background

Periodontitis is among the most prevalent human diseases in the world. Estimates place the number of affected at 740 million people globally. It has a heavy disease burden, affecting systemic health, nutrition, quality of life and self-esteem, and poses big socio-economic impacts and healthcare costs [1, 2].

It is well established that early diagnosis and regular maintenance are of paramount importance in managing the presence and progression of Periodontitis [3]. The current Classification of Periodontal and Peri-Implant Diseases and Conditions classifies Periodontitis in stages and grades. Stages go from 1 to 4 and classify the severity, extent and complexity of a patient’s Periodontitis, while grades go from A to C and estimate the future risk of progression, responsiveness to treatment and impact on systemic health [4]. The classification establishes that a thorough radiographic evaluation of the patients’ periodontal condition is necessary for diagnosis and maintenance, with the stage of radiographic Periodontal Bone Loss (PBL) serving as an index for determining stage and grade [4]. Table 2 depicts PBL stages in the current classification.

Commonly used radiographs for measuring PBL are periapical, bitewing and panoramic techniques [5]. While periapical and bitewing radiographs provide detailed information about individual teeth, the panoramic radiograph offers a broader overview of the patients’ general condition. In patients with generalized Periodontitis, the panoramic radiograph is recommended as an initial and periodic screening method due to its wide-ranging focus, along with other desirable characteristics, such as low radiation exposure, high accessibility, and the amount of information provided [5].

Meanwhile, academic publications about Artificial Intelligence (AI) applied to dentistry are rapidly increasing [6]. A simple and helpful definition of AI is a machine capable of behaving intelligently, imitating a human cognitive ability such as listening, speaking or observing [7]. Various AI techniques and applications have been proposed in the area, with a primary focus on using Machine Learning (ML) models for automating radiographic analysis [8–10]. Among ML paradigms, Supervised Learning studies algorithms that learn by means of a labelled dataset, optimizing their internal structure to achieve the best possible performance on new data points that are “similar” to those observed at training [7]. For automating radiographic analysis, a model can be trained with a labelled dataset containing instances of a particular pathology. Afterwards, the trained model is compared to an expert criterion, usually a human specialist or another well-established diagnostic test, and various indices are calculated to determine the model’s performance. Comparison to an expert criterion allows for a point of comparison for performance indices (such as sensitivity, precision or F1-score) to be interpreted from, given that they do not possess integrated categorical scales or cut-off points for describing their values [7]. The successful automation of multiple oral diseases has been achieved with real time diagnosis and acceptable performance, some having reached a product and service phase [11–13].

Considering the high prevalence of Periodontitis and the need for radiographic evaluation in its diagnosis and maintenance, using tools such as ML models capable of automating radiographic analysis could prove beneficial to currently practiced workflows. ML models applied to PBL have already been reported, although, to the best of our knowledge, never in the Chilean population [8].

The aim of this study was to determine the efficacy of an ML model for automatically measuring Periodontal Bone Loss (PBL) in panoramic radiographs by comparing it to dentists.

## Methods

We collected 500 panoramic radiographs from two distinct populations. On one hand, 250 radiographs were obtained from a particular, non-representative Chilean population composed of patients treated at the Department of Periodontics from Universidad de los Andes at San Bernardo, Santiago-Chile, during the academic year 2021. On the other hand, 250 radiographs belonged to a publicly available dataset from Tufts University, Massachusetts-USA. The authors voluntarily shared this dataset with us, and the access link can be found in the references [14]. For identification and anonymity, each radiograph was assigned a code composed of a number and its origin population (e.g., SB001, USA 001). The Chilean subset was obtained from the local electronic database, and the USA subset was obtained from the published dataset. Both sets of radiographs were sampled at random respectively from a larger population. No other demographic characteristics were considered or recorded from either population (i.e. age, gender, ethnicity or socio-economic status). Two types of radiographs were excluded from the study: those from edentulous patients and those from patients with temporary/mixed dentures.

All radiographs were manually labelled to add explicit information that would act as the model supervision. This labelling task was done by the first author, an undergraduate student, using Label-Studio® on a MacBook Air® 2015 (Intel Core® i5 CPU, macOS Sierra 10.12.6 OS). The labeller received spoken and written instructions pertaining to the correct PBL measuring technique, obtained from the scientific literature [15]. These instructions were given by a radiologist with more than 5 years of experience. An initial training phase was performed, where the labeler exercised on an initial batch of non-used panoramic radiographs and double checked the labeled PBL points and bounding boxes against the radiologist’s experience, receiving feedback in cases of error. This training process comprised a total of 6 hours spread throughout 1 week.

**Fig. 1:**
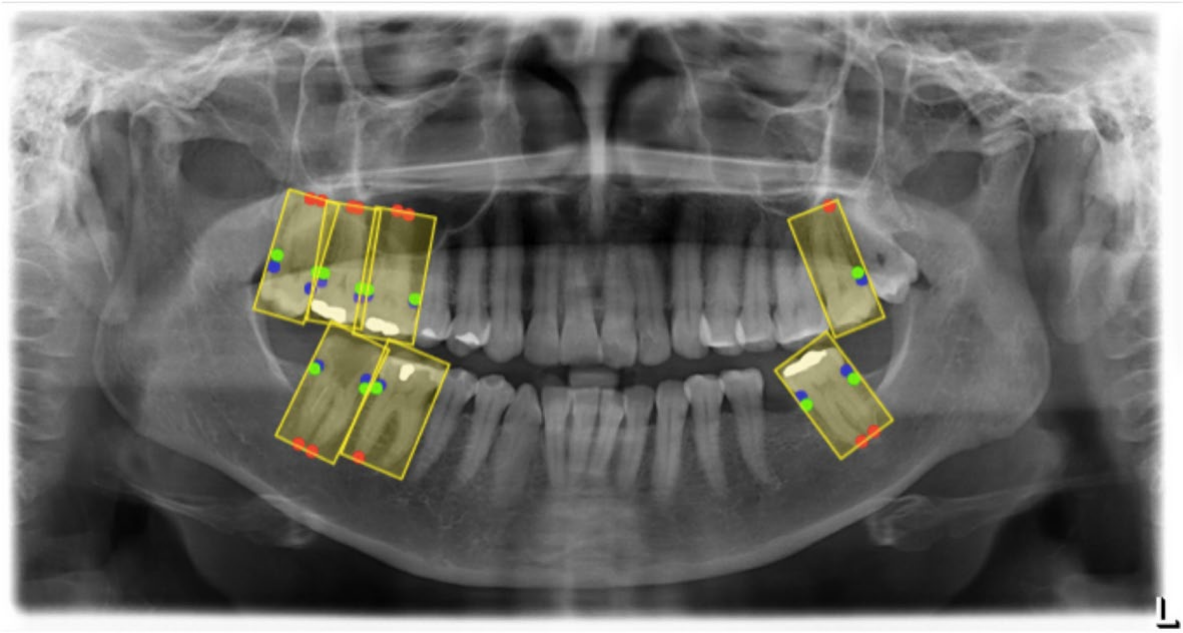
Data labeling process. Legend: “In yellow, bounding boxes were labelled to contain molar teeth. Blue dots= CEJ. Green dots= alveolar crest. Red dots= root apex”

The labelling process consisted of two tasks: creating bounding boxes containing teeth and identifying key points for radiographic PBL calculation on each tooth. As an inclusion criterion, only molars were considered for this study. Firstly, a bounding box was drawn to contain each molar in every panoramic radiograph. Then, key points were labelled to represent the necessary radiographic points for calculating PBL, which included: 1. Cementoenamel Junction (CEJ), 2. the portion of Alveolar Crest closest to the ligament space, and 3. the Root Apex. This accounted for three points per anatomical side of a given molar (mesial and distal), totaling six key points and one bounding box per labelled molar. As an exclusion criterion, it was established a priori that key points would only be labelled when all three of the necessary radiographic points for calculating PBL would be “easily identifiable” in one anatomic side simultaneously. If any of the three radiographic PBL points was deemed obstructed by the labeller, none of the other two were labelled, even if plainly visible. Figure 1 shows the data labelling process, and Table 1 shows the most frequently observed obstructive factors during data labelling.

**Table 1:** Obstructive radiographic points

Obstructive Factor	Affected Key Points
Dental Crowding	CEJ, Crest
Maxillary Sinus Cortical	Crest, Apex
Overexposure	CEJ, Crest, Apex
Underexposure	CEJ, Crest, Apex
Dental Caries	CEJ
Restorations	CEJ
Root Resorption	Apex

The labeller was calibrated by calculating its Intraclass Correlation Coefficient (ICC). For this, he was asked to label 20 additional radiographs, 10 from each population, and after 1 month, to label them again. A third researcher then compared both sets of measurements and ICC was calculated.

**Fig. 2:**
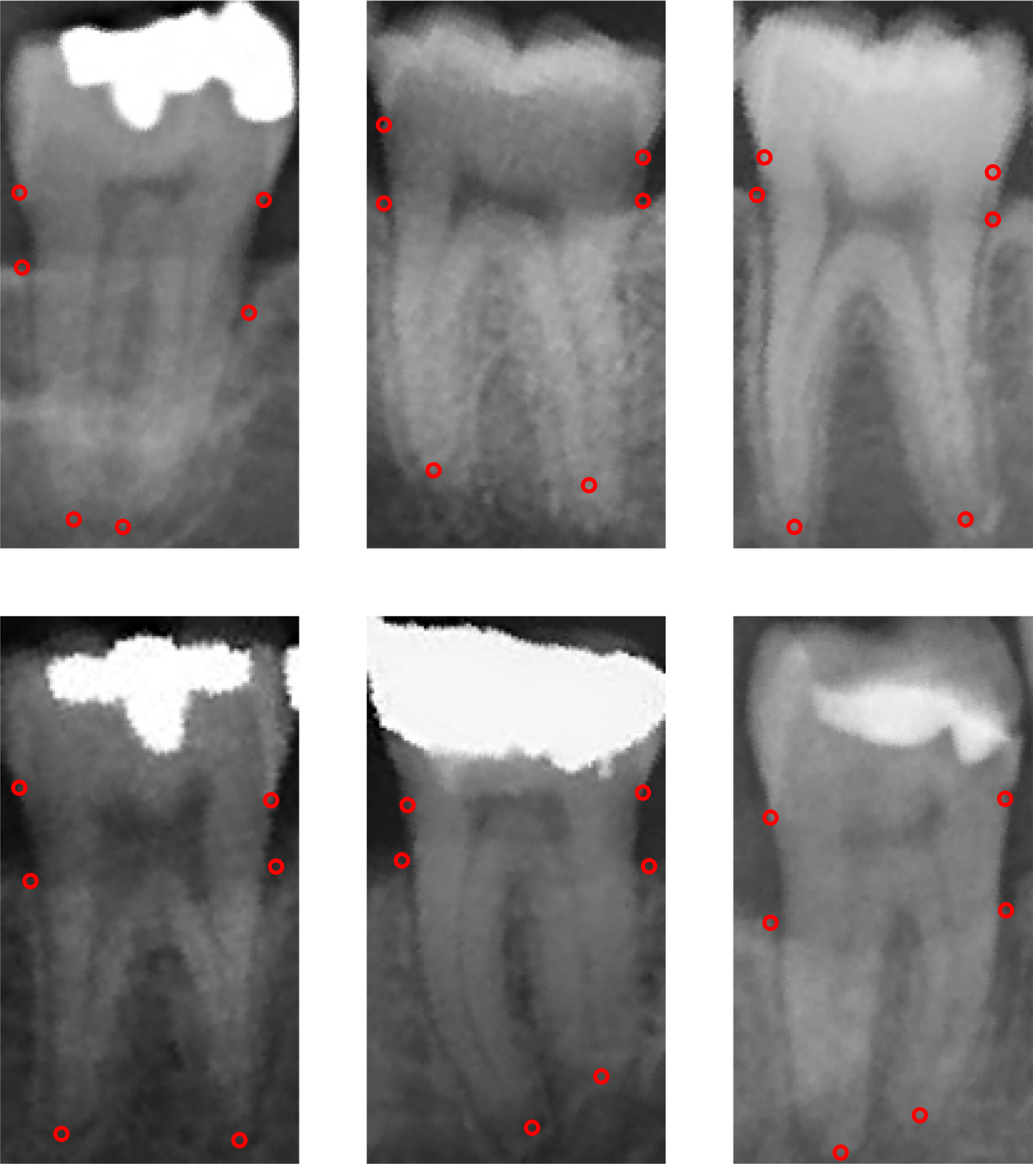
Segmented molar images with PBL points. Legend: “Sample of the resulting segmented molar images (1970) after data processing of labelled panoramic radiographs. Each molar image contains the necessary radiographic points for PBL calculation”

Once the dataset was completely labelled, an algorithm automatically segmented all bounding boxes from each radiograph. This created 2010 rectangular images containing 1 molar along its labelled radiographic points necessary for PBL calculation [Fig. 2]. Afterwards, a final processing step was carried out: all upper molar images were inverted by 180°. This was done so that the model could process, both during training and inference, all the root apex points as located in the lower portion of the rectangular images. This would help with the stability and convergence of the training process, a key prerequisite in producing a useful model.

With the newly segmented 2010 rectangular images, the data was partitioned to create training and testing datasets. Forty images were sampled at random to be later used for testing purposes. These images had their labeled PBL points removed. The remaining 1970 rectangular labelled images comprised the training dataset, which was further divided into 80% (1576) for training and 20% (394) for internal validation. These images were used with their PBL points present.

**Fig. 3:**
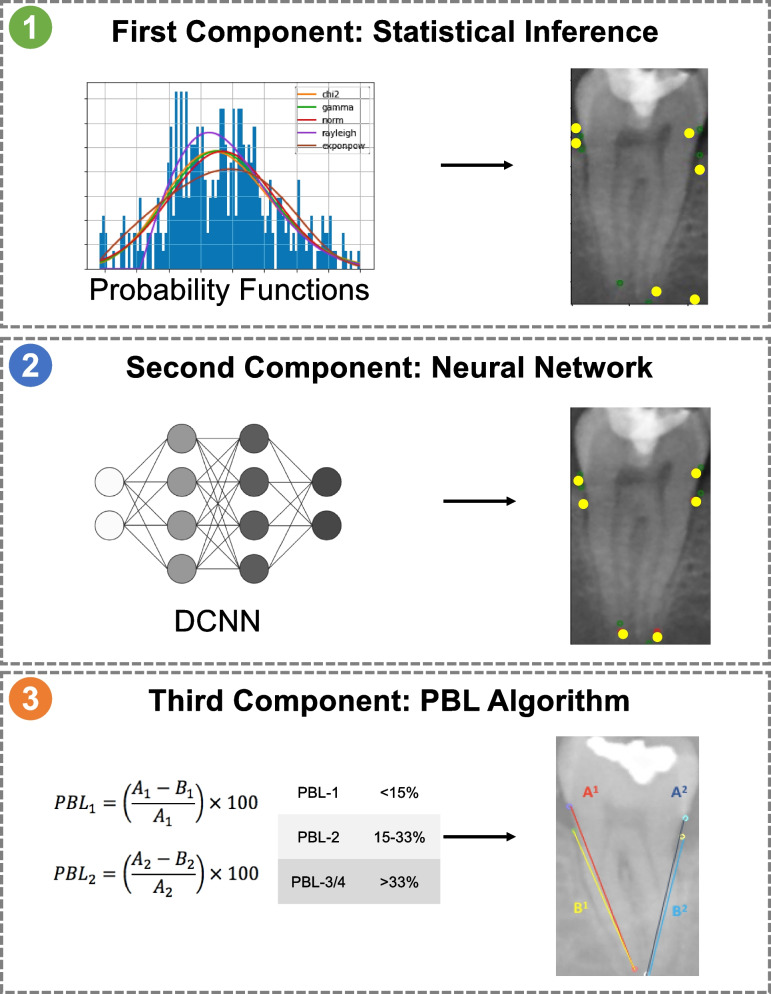
Model components and workflow. Legend: “1. The first component samples probability functions to create a statistical, yet inaccurate, prediction of PBL points in a molar image. 2. The second component is a deep convolutional neural network that further refines the first component’s prediction. 3. The third component calculates PBL percentage and stage from the second component’s prediction”

As an overview, our ML model was structured into three components, which were executed sequentially. The first two components aimed to create a vector of six ordered pairs that would represent the exact pixels where each of the six radiographic points necessary for calculating PBL would be located in a rectangular molar image. The third and final component would take the generated vector and express it as two lines (one for representing root length and one for bone height) to further calculate PBL percentage and stage. More specifically, the first component created an initial prediction of PBL points by means of statistical inference techniques. The second component refined the initial prediction by using a Deep Convolutional Neural Network (DCNN). The third and final component used a rule-based algorithm that translated the second component’s refined prediction into PBL percentage and stage. Figure 3 illustrates the model’s components and workflow.

The first component created an initial prediction by determining the probability functions that best adjusted to the information contained in the training dataset. This process was performed for each of the 12 coordinates which constituted the six ordered pairs in each molar image (representing the CEJ, alveolar crest, and apex of both mesial and distal aspects). The result was two probability functions per ordered pair, totaling in 12 distinct probability functions. When each function was sampled simultaneously, the result was an initial, purely statistical prediction of the radiographic points’ location. This sampling process was repeated 25 times per image, generating additional examples with slightly different predictions each time. This approach allowed for data augmentation, which created a total number of 10,050 predictions that are considered as input in the next stage. This component utilized the package “fitter” 1.4.1 [16].

The second component consisted of refining the prediction of the first component by conditioning it to the visual content of each molar image. For this purpose, we used a Deep Convolutional Neural Network (DCNN) with a fixed number of input/output pairs. The input of the DCNN consisted of paired rectangular images of molars along with their predictions from the first component, while the output was the real annotation of the labelled training dataset. Overall, this component had a total of 10,050 input/output pairs. The network was defined and trained with Google TensorFlow Keras® version 2.10.0. The architecture of the DCNN followed Xception Network’s design [17]. The loss function to minimize was the mean squared error between a 12-dimensional vector emitted by the second component (DCNN) and the real vector (training dataset). The DCNN was trained for five epochs controlled with early stopping and an Adam optimizer with a learning rate of 0.001 [18].

Hyperparameters included the number of filters in CNN layers, activation function type and number of output neurons in the last layer. These hyperparameters were manually adjusted based on observed training dynamics (i.e., final loss value). The batch size was set to 32, and dropout was applied to all layers except the final layer at a rate of 0.5 (i.e., a 50% chance of turning off each neuron during the forward pass). No other regularization parameters were used apart from dropout. Internal model validation was performed during training by computing the loss and accuracy on a validation dataset consisting of 20% of the total data in the training dataset held out for this purpose.

**Fig. 4:**
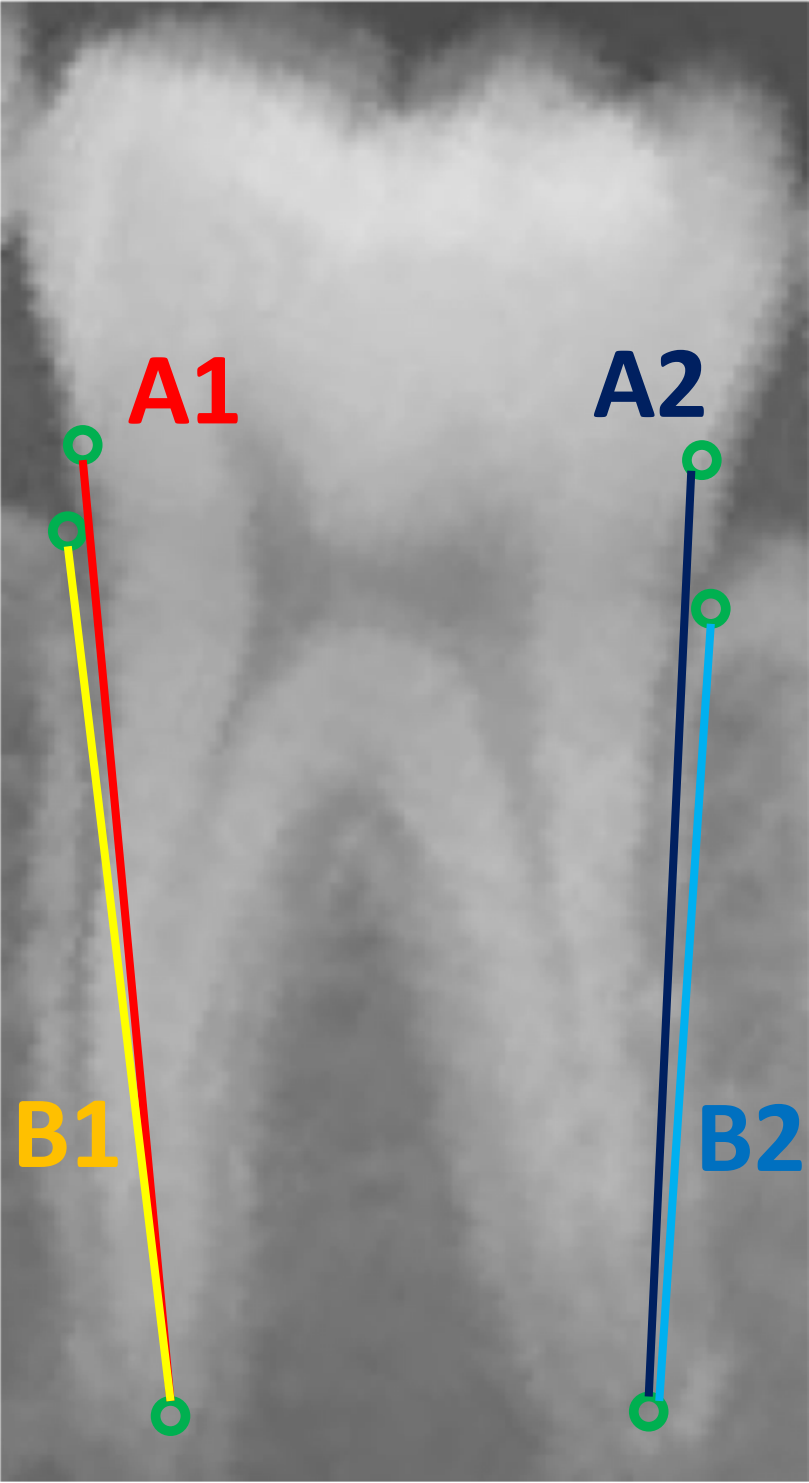
Radiographic PBL calculation. Legend: “Lines drawn between CEJ and root apex points represent root length: A1= left root length, A2= right root length. Lines drawn between the alveolar crest and root apex points represent bone height: B1= left bone height, B2= right bone height. PBL was calculated as follows: PBL 1 (%) = (A1-B1/A1) *100. PBL 2 (%) = (A2-B2/A2) *100”

The third and final component was a rule-based algorithm for calculating PBL percentage and expressing it as a stage based on the prediction made by the second component. This algorithm generated two lines connecting the ordered pairs that represented a molar’s root length (established between Apex and CEJ points) and its periodontal bone height (established between Apex and Alveolar Crest points), as illustrated by Fig. 4. Afterwards, it automatically calculated the resulting quotient and expressed it as a percentage. For each resulting percentage, the algorithm assigned the corresponding PBL stage according to the classification proposed by the Classification of Periodontal and Peri-Implant Diseases and Conditions, found in Table 2.

**Table 2:** PBL calculation and stage according to the Periodontal and Peri-Implant Diseases and Conditions Classification [4]

PBL Amount (%)	Stage
< 15%	1
15–33%	2
> 33%	3/4

Regarding the computational resources needed for creating and training the model, a cloud server from Google Colab was used, equipped with an Nvidia Tesla® P100 GPU with 16GB VRAM and two Intel Xeon CPUs running at 2.30Ghz with a total of 13GB of RAM.

Once trained and validated, the model was compared to five human participants in a standardized test. These participants were two radiologists, two periodontists, and one general dentist. It was decided a priori that the two radiologists would collectively represent the correct answers of the test (ground truth), while both periodontists and the general dentist would serve as additional comparisons for the model. A third radiologist was consulted for resolution in cases of differing responses between radiologists. All participants were registered as individual national healthcare providers in the Chilean Health Superintendence of the National Health Ministry (MINSAL). Additionally, the amount of time since the date of degree emission for each of the participant’s profiles was recorded. A document of informed consent was previously signed voluntarily by each participant. This document explained the nature of the study, its purpose, implications for participants and measures for assuring confidentiality.

The test was conducted in a classroom located at the Universidad de los Andes campus in San Bernardo, Santiago, Chile. All human participants performed the experiment at the beginning of the workday morning, immediately after arrival to the clinical center and without any previous form of work. This was done to ensure absence of physical and mental fatigue during the experiment across participants. Each participant performed the experiment individually, alone in the classroom save for an experiment supervisor and with all classroom lights turn off. As mentioned, the test comprised 40 unlabeled rectangular molar images displayed on a 13.3-in. monitor with a resolution of 1440 × 900. Participants were previously instructed on the expected technique for measuring PBL, involving all three radiographic PBL points and the subsequent method of percentage calculation and stage assignment. The test instructions were that for each molar image, the participants had to measure the percentage of PBL and determine the corresponding periodontal stage on both the right and left sides of each molar image. Participants were also provided a ruler and white sheet of paper alongside the test answer sheet. Before beginning the test, the participants openly stated that they understood the requested task. The time taken by each participant was measured in seconds. Subsequently, the model was run on all molar images, and the time taken was recorded.

Data analysis for binary classification was conducted. Responses made by each participant, including the model, were categorized based on diagnostic indices (True Positive, True Negative, False Positive, False Negative) in relation to the responses of both radiologists. These indices were calculated considering each possible answer class (PBL stage 1, 2, and 3/4) as separate binary tests. Table 3 displays the expression of the diagnostic indices for each PBL class.

**Table 3:** Expression of diagnostic indices for each PBL class

Stage 1				Stage 2				Stage 3			
Truth		Test Answer		Truth		Test Answer		Truth		Test Answer	
		1	2,3			2	1,3			3	1,2
	1	TP	FN		2	TP	FN		3	TP	FN
	2,3	FP	TN		1,3	FP	TN		1,2	FP	TN

Legend: *TP* True Positive, *TN* True Negative, *FP* False Positive, *FN* False Negative.

Performance metrics were computed for each participant in each stage. The calculated metrics were sensitivity, specificity, recall, precision and F1-score. Performance averages were also determined, including macro-, weighted-, and micro-averages. All acquired data was tabulated and analyzed in spreadsheets using Microsoft Excel®.

This study was approved by the Research Committee of the Dental Faculty and the Scientific Ethics Committee of Universidad de los Andes. All patients from the Chilean population whose radiographs were used in the study voluntarily signed an Informed Consent document at the time of treatment.

## Results

The ICC value for the data-labeling observer was 0.91. The participants’ years of experience can be found in the Supplemental Files. Regarding the test results provided by both radiologists, PBL stage 1 was identified in 57.14% of the instances, PBL stage 2 in 35.71%, and PBL stage 3/4 in 7.14%. The average value for each PBL stage was 10.58% for stage 1, 24.84% for stage 2, and 46.66% for stage 3/4.

Table 4 displays the recorded time for each participant. On average, radiologists took 23.2 minutes to complete the test, while controls took 26.9 minutes. The overall human average (radiologists and controls) was 25.4 minutes. In contrast, the model answered the test in 0.93 seconds.

**Table 4:** Time obtained by each participant in standardized test

Participant	Time (m/s)	
Radiologist 1	23.38	m
Radiologist 2	23	m
Periodontist 1	31.48	m
Periodontist 2	18.78	m
General Dentist	30.31	m
AI Model	0.93	s

Legend: “*m* minutes, *s* seconds”

For PBL stage 1, the model obtained the second-highest sensitivity of the group (0.5), following periodontist 2 (0.75), the lowest precision (0.26), recall (0.42) and specificity (0.39) of the group, and the third highest value for F1-score (0.34). For stage 2, the model obtained the second-highest sensitivity of the group (0.4) after Periodontist 2 (0.46), the third-highest value for precision (0.17) and F1 score (0.24), and the lowest value for recall (0.53) and specificity (0.56). Regarding stage 3/4, the model obtained values of 0 for sensitivity, precision and F1 score, although the highest value of the group for recall (0.96) and specificity (1.00). Periodontist 2 achieved the highest indices in every stage. All values obtained by every participant on each performance metric are listed in Table 5.

**Table 5:** Performance metrics obtained by each participant

	Stage 1	Stage 2	Stage 3/4
**Sensitivity**			
General Dentist	0.417	0.333	1.000
Periodontist 1	0.208	0.000	1.000
Periodontist 2	0.750	0.467	1.000
AI Model	0.500	0.400	0.000
**Specificity**			
General Dentist	0.696	0.723	0.870
Periodontist 1	0.804	0.769	0.753
Periodontist 2	0.804	0.954	0.909
AI Model	0.393	0.569	1.000
**Recall**			
General Dentist	0.613	0.650	0.875
Periodontist 1	0.625	0.625	0.763
Periodontist 2	0.788	0.863	0.913
AI Model	0.425	0.538	0.963
**Precision**			
General Dentist	0.370	0.217	0.231
Periodontist 1	0.313	0.000	0.136
Periodontist 2	0.621	0.700	0.300
AI Model	0.261	0.176	0.000
**F1 Score**			
General Dentist	0.392	0.263	0.375
Periodontist 1	0.250	0.000	0.240
Periodontist 2	0.679	0.560	0.462
AI Model	0.343	0.245	0.000

Concerning macro average, the model yielded the lowest values for sensitivity (0.3), specificity (0.65), precision (0.14) and recall (0.64), while ranking third in terms of F1 score (0.19). Periodontist 2 obtained the highest values for all five metrics.

Regarding weighted average, the model obtained the second-highest sensitivity (0.23), tied to General Dentist, the third-highest value in precision (0.11) and F1 score (0.15), and the lowest in recall (0.26) and specificity (0.26). Periodontist 2 obtained the highest values for all five metrics.

Finally, about micro-average, the model obtained the second-highest sensitivity (0.42), tied to General Dentist, and the third highest value for precision (0.22) and F1 score (0.29). Once again, Periodontist 2 obtained the highest values for all three metrics. The values obtained by each participant on macro-, micro-, and weighted averages are listed in Table 6.

**Table 6:** Performance averages obtained by each participant

	Sensitivity	Precision	F1 Score	Specificity	Recall
Macro-Average					
General Dentist	0.583	0.273	0.343	0.763	0.713
Periodontist 1	0.403	0.150	0.163	0.775	0.671
Periodontist 2	0.739	0.540	0.567	0.889	0.854
AI Model	0.300	0.146	0.196	0.654	0.642
Weighted-Average					
General Dentist	0.230	0.160	0.180	0.380	0.340
Periodontist 1	0.100	0.100	0.080	0.410	0.330
Periodontist 2	0.350	0.330	0.330	0.450	0.430
AI Model	0.230	0.110	0.150	0.260	0.260
**Micro-Average**					
General Dentist	0.429	0.286	0.343		
Periodontist 1	0.190	0.151	0.168		
Periodontist 2	0.667	0.571	0.615		
AI Model	0.429	0.225	0.295		

## Discussion

The model proposed in this study aimed to automate the diagnosis of radiographic Periodontal Bone Loss (PBL) using a Deep Convolutional Neural Network (DCNN), which managed to do with acceptable performance and real-time diagnosis.

The dataset used for this study was particularly small and below the recommended size for training models similar to ours [19]. Study design determinations were made to ensure the best possible performance in the context of limited data access. The first determination was the use of two distinct populations. While the Chilean population of periodontal patients was the original subject intended to be studied, a second population, in the form of a publicly available dataset from Tufts University, was included due to the difficulty of obtaining a sufficiently large dataset from the original population alone. This decision might have consequences for the model’s performance, as it has been proposed in the relevant literature that the dataset should come from the population for which an AI is intended to be used [20]. We propose that any detrimental effects in the model’s capability to generalize in the used Chilean population is offset by the fact that including a second population enables the existence of the model in the first place. It is also possible to consider finetuning the weights as additional Chilean data is made available.

A clear limitation is that neither of the populations used in this study were representative in nature. The Chilean population originated from a subset of periodontal patients from a specialized care center. Additionally, the demographic characteristics of both populations remained unclear. This is relevant, given that the used dataset largely determines a model’s learned patterns. Given the non-representative nature of the data, the model’s performance retains validity only in relation to these two specific populations, while its performance on external populations remains undetermined. On the same note, considering that the Chilean population is from a subset of periodontal patients, there is a considerable chance of it being skewed towards over representing periodontally compromised patients over healthy ones. In the context of automating a radiographic pathology, achieving a balanced dataset where all ranges of a given disease are uniformly represented is of paramount importance.

The number of radiographs included in the study (500) represented the maximum amount that the researchers managed to manually label during the study’s allocated time frame. The size of the used dataset constitutes an important limitation on both model training and evaluation procedures, given the widely known fact that ML models perform better, all other conditions being unchanged, if the dataset is more extensive [17].

The second significant determination was using panoramic radiographs and molars as the units of study. It was determined in advance that a limited amount of data should, in turn, be as standardized as possible to promote effective learning by the model. Panoramic radiographs were chosen as study technique due to their inherently standardized capture method, which involves taking images around a fixed occlusal block [21]. Similarly, it was decided to avoid including anterior teeth to regularize anatomical features across data points, which limits performance and validity in teeth other than molars [21].

Another limitation was using a single observer during data labelling, which manifested in the form of Obstructive Factors [Table 1], where the observer could not be sure of the precise location of certain radiographic points. Having two or more observers responsible for data labelling would solve this issue and reduce bias. When faced with visually challenging points, multiple observers could make collective decisions, which would offer two advantages: firstly, there would be no excluded radiographic points, therefore utilizing data more efficiently, and secondly, it would enhance the model’s performance by providing information about precisely those visually demanding points. The use of a sole observer helps explain the performance observed. Nonetheless, the obtained ICC value (0.91) argues for a well-instructed and calibrated undergraduate student.

Still, the main limitation regarding this model’s clinical validity is that it cannot measure PBL on unprocessed panoramic radiographs. As previously reported, the model can only measure PBL on processed cuts containing molar images from a panoramic radiograph. While it was attempted to have it perform this task on unprocessed radiographs using another CNN, this still needs to be achieved. This lack of success explains the first component as a workaround for this issue. Considering that this pre-processing phase encompassed between 6 to 8 hours of computing, this limitation would have to be resolved for creating a truly automated model. Therefore, a clear direction for future work would be to train a CNN to automatically segment molar cuts from a panoramic radiograph, allowing the existing model to then measure the amount of PBL in those cuts. Such an approach would aid in creating a clinically useful model.

Another investigative line worth considering would be to create a model for automating PBL measurement based on regression equations, that could then be trained and compared to dentists in a binning basis instead of a categorical stage basis. This, in turn, would allow to further study and consider the level of similarity between the model and a human standard in a percentage-by-percentage manner, something that our proposed method cannot perform.

Regarding the standardized comparison test, 40 images were drawn at random. This number was chosen as it appeared to be representative enough of the population of study, yet not high enough to start imposing fatigue on human participants. As mentioned, this random sampling resulted in an unbalanced testing set, given the expert criteria of both radiologists identifying most instances as light to moderate PBL (Stage 1 = 57.14%, Stage 2 = 35.71%) and the vast minority as severe PBL (Stage 3/4 = 7.14%). We suspect this is because the training dataset did not contain many instances of PBL stage 3/4 to begin with.

The data was analyzed as a binary classification system. This means that even though the model was tested on a question which has a multi-class answer (i.e., there is PBL, and it is one of three possible choices: 1, 2 or 3/4), each of the possible answer classes were considered as separate and independent tests (i.e., there’s three types of tests, one per each PBL class, and one can either identify it successfully or not). This was done so that the diagnostic indices of true positive, true negative, false positive and false negative could be calculated in the first place, given that they can only be expressed in binary form. This, in turn, created sets of diagnostic indices for each PBL class on each participant [Table 3].

As previously stated, an assumption was made a priori that both radiologists would collectively represent the ground-truths of the study over that of other participants (periodontists and general dentist). This assumption was made on the need for determining a set of answers to be considered as known true data points, or as ground-truths. Given the studied variable of measuring radiographic PBL and considering the specialty of Radiology to be the most closely related to the analysis of radiographic images, it was decided to consider the radiologists’ answers as ground-truths over that of the both periodontists’ and general dentist.

Afterwards, performance metrics were calculated (sensitivity, specificity, precision, recall and F1-score). Sensitivity and precision both express information about the success rate in positive answers (i.e., the capacity to identify presence of disease), on the contrary, specificity informs about the success rate in negative answers (i.e., the capacity to identify absence of disease), recall informs about the rate of success of positive and negative answers at the same time, and F1-Score is the harmonic mean between sensitivity and precision. Moreover, given the unbalanced testing set, averages were further calculated. Given the data, both weighted- and micro-averages stand as the most precise indices, as they take into consideration the observed frequency of each answer class and, therefore, describe an unbalanced dataset more precisely. On the contrary, macro-average assumes all classes as equally prevalent.

Regarding the model’s performance, specifically around the time variable, one thing is concluded: the model was exceedingly faster than the human participants, as it achieved real time diagnosis in practical terms, diagnosing 40 teeth in 0.93 s, or 0.02 s per tooth. Further down the implications of this result will be discussed.

Regarding the model’s performance for measuring PBL, it obtained an acceptable performance for detecting PBL stages 1 and 2, or light to moderate, as it obtained values of 0.23 and 0.29 for weighted sensitivity and F1-score, respectively. It expressed a slight tendency towards over-diagnosing PBL stage 2, as it obtained a value of 0.17 for precision in said stage. Lastly, it proved incapable of detecting PBL stage 3/4, or severe PBL, as it obtained values of 0 for both sensitivity and F1-score in this stage.

Compared to the human controls, the model obtained a similar overall performance against the General Dentist and Periodontist 1, given the comparable values obtained by the model on the weighted and micro averages of sensitivity, precision and F1 score [Table 6]. However, it obtained a worse performance when compared to Periodontist 2 and both Radiologists, given the lower values obtained by the model in the weighted and micro averages of sensitivity, precision and F1 score. When considering the performance obtained between human controls (periodontists and general dentist), there was a noticeable better performance from Periodontist 2 across all performance indices, which can be explained by the considerably larger trajectory from this participant both as a DDS and as a periodontist (Supplemental files).

As previously noted, weighted- and micro-average values are affected in direct proportion to an observed class’ prevalence. Therefore, the model’s performance was not significantly affected by its inability to measure PBL stage 3/4, as this class expressed a very low prevalence. However, the fact remains: not being able to detect the most severe cases of a pathology constitutes a serious limitation. Another direction for future research would be to create a new dataset composed exclusively of instances of PBL Stage 3/4 to strengthen the models’ performance on this stage.

The results of this study allow for multiple implications. Firstly, we have demonstrated that the method used here is overall data efficient. In the context of Machine Learning algorithms like Deep Convolutional Neural Networks, a dataset of 500 original images (panoramic radiographs) represents a low number, which can nevertheless work thanks to the utilization of data augmentation techniques and study design resolutions to increase the amount of useful data points [17].

A second implication is the demonstrated capacity to automate the measurement of PBL in molars from panoramic radiographs using Machine Learning. Considering Periodontitis’ prevalence, not only in Chile but also globally, coupled with the known significance of early diagnosis and periodic monitoring for at-risk and affected populations [3], the value of this model starts to become evident, as it could help automate the radiographic analysis of a massively prevalent condition, helping diminish both time and human resources needed in the currently practiced workflows.

Moreover, the automation of other radiographic pathologies has already been reported, such as interproximal caries, periimplantitis, and even tumours and cysts of the jaw [9, 10, 22]. All these conditions rely heavily on early diagnosis and maintenance to facilitate disease prevention and arrest progression, both aimed at conserving as much healthy tissue as possible. Looking ahead, the development of ML models capable of simultaneously automating the diagnosis of multiple pathologies shows excellent promise. Such models would significantly enhance clinical workflows, enabling practitioners to work more swiftly and precisely, diminishing fatigue and, as research shows, increasing sensitivity to certain pathologies [10]. Further studies are needed to determine the nature and extent of these models’ effect on healthcare workflows and services.

This is not to say that there are no obstacles to work through, as proposed, there are multiple challenges to be addressed, mainly: 1. Ensuring data protection and security, given the need for datasets composed of sensitive patient information. 2. Gathering and producing sufficiently large and standardized datasets, as they are needed for creating differentiated AI models. 3. Creating and inserting clinically useful models that are transparent, reliable, and unbiased [23]. Resolving these challenges will ensure the long-term success of healthcare-applied AI models that make dental care faster, better, and more widely available.

One final future direction would be to apply this architecture to different features found in and around the dental structure in panoramic radiographs, such as caries, dental restorations, periapical lesions, type of bone defect present in PBL (i.e., vertical or horizontal defects), and presence of furcation lesions.

## Conclusions

The Machine Learning model developed in this study achieved acceptable performance and real-time diagnosis for measuring Periodontal Bone Loss (PBL) in cropped images of molars from panoramic radiographs. In the future, using similar tools could improve workflows for multiple oral pathologies with radiographic manifestations.

### Supplementary Information

**Additional file 1: Supplementary Table 1. **Years of Experience of Human Participants from Standardized Test.

## Data Availability

The dataset used for conforming the USA population is available from the corresponding author on reasonable request in the following link (also available in the References section): http://tdd.ece.tufts.edu/. Both the code used for the Machine Learning model and the Excel Spreadsheets used for data analysis can be requested to the corresponding author at dortuno@uandes.cl. The Chilean population’s radiographs represent private information for which we have no publication clearance.

## Abbreviations

PBL: Periodontal Bone Loss
AI: Artificial Intelligence
ML: Machine Learning
CEJ: Cemento-enamel junction
ICC: Intraclass Correlation Coefficient
DCNN: Deep Convolutional Neural Network
VRAM: Video Random-access Memory
RAM: Random-access Memory
GPU: Graphics Processing Unit
CPU: Central Processing Unit
TP: True Positive
TN: True Negative
FP: False Positive
FN: False Negative
