# Supplementary material for: “Determining the efficacy of a machine learning model for measuring periodontal bone loss”
Source: BMC Oral Health. 2024 Jan 17;24:100. doi: 10.1186/s12903-023-03819-w (PMC10792795; doi:10.1186/s12903-023-03819-w)
Supplement: Supplementary file 1 — Additional file 1: Supplementary Table 1. Years of Experience of Human Participants from Standardized Test. [file 12903_2023_3819_MOESM1_ESM.docx]

| **Operator** | **Number of years since DDS degree** | **Number of years since specialty degree** |
| --- | --- | --- |
| Radiologist 1 | 3.83 | 0.83 |
| Radiologist 2 | 7.83 | 5.75 |
| Periodontist 1 | 12.58 | 8.92 |
| Periodontist 2 | 21.83 | 14.58 |
| General Dentist | 0.83 | - |
|  |  |  |
|  |  |  |

**Supplementary Table 1: Years of Experience of Human Participants from Standardized Test.**

Legend: DDS = Doctor of Dental Surgery.
